# Cell Therapy in Multiple Sclerosis: Clinical Advances, Limitations, and Future Perspectives from Clinical Studies—A Systematic Review

**DOI:** 10.3390/pharmaceutics18010030

**Published:** 2025-12-25

**Authors:** Ola Mohamed Fathy Kamal, Doddy Denise Ojeda-Hernández, Belén Selma-Calvo, Marina García-Martín, María Teresa Larriba-González, Lucia Martin-Blanco, Jordi A. Matias-Guiu, Jorge Matias-Guiu, Ulises Gomez-Pinedo

**Affiliations:** 1 Laboratorio de Neurobiología y Terapias Avanzadas, Instituto de Neurociencias, Instituto de Investigación Sanitaria San Carlos (IdISSC), Hospital Clínico San Carlos, 28040 Madrid, Spain; aolakamal@gmail.com (O.M.F.K.); doddydenise@gmail.com (D.D.O.-H.); belselma@ucm.es (B.S.-C.); maring80@ucm.es (M.G.-M.); mlarri01@ucm.es (M.T.L.-G.); lumart26@ucm.es (L.M.-B.); jordimatiasguiu@hotmail.com (J.A.M.-G.); matiasguiu@gmail.com (J.M.-G.); 2 Servicio de Medicina Física y Rehabilitación, Hospital Central de la Defensa, 28047 Madrid, Spain; 3 Servicio de Neurología, Instituto de Neurociencias, Instituto de Investigación Sanitaria San Carlos (IdISSC), Hospital Clínico San Carlos, Universidad Complutense de Madrid, 28040 Madrid, Spain

**Keywords:** multiple sclerosis, cell therapy, clinical trials, stem cells, immunomodulation, neuroregeneration

## Abstract

**Background:** Multiple sclerosis (MS) is a chronic autoimmune demyelinating disease of the central nervous system (CNS), characterised by inflammation, demyelination, and progressive neurodegeneration. Although current disease-modifying therapies (DMTs) can reduce relapse rates and inflammatory activity, they rarely stop long-term progression or repair neurological damage. In recent years, cell-based therapies have emerged as promising approaches to promote immune regulation and neuroregeneration in MS. **Methods:** This review summarises the current clinical evidence from studies in humans investigating cell-based treatments for MS, including autologous haematopoietic stem cell transplantation (AHSCT), mesenchymal stem cells (MSCs), and neural stem or progenitor cells (NSCs). A systematic literature search was performed using PubMed, Scopus, and ClinicalTrials.gov, focusing on human clinical trials that met specific inclusion criteria. **Results:** Prevailing findings show that AHSCT provides the most consistent benefit, achieving long-term immune reconstitution and remission in patients with highly active relapsing–remitting MS (RRMS), although it carries procedural risks. MSC therapies have demonstrated good safety and biological activity, especially when delivered intrathecally (IT) in progressive MS, though clinical results remain variable. **Conclusions:** NSC-based treatments are still at an early stage of clinical research but show potential for CNS repair. The main limitations across studies include differences in protocols, small sample sizes, and short follow-up periods. Further large-scale, randomised controlled trials are needed to confirm long-term efficacy, define optimal delivery methods, and establish standardised clinical protocols.

## 1. Introduction

Multiple sclerosis (MS) is a immune-mediated disorder of the central nervous system (CNS) characterized by inflammatory activity, demyelination, gliosis, and progressive neuroaxonal degeneration. Affecting more than 2.8 million people worldwide, MS remains one of the most common causes of non-traumatic disability in young adults and generates a substantial socioeconomic burden due to long-term neurological impairment, early workforce loss, and elevated healthcare needs. Clinically, MS manifests in several forms—relapsing–remitting (RRMS), secondary progressive (SPMS), and primary progressive (PPMS)—each with distinct biological and clinical features [1,2,3,4,5,6,7,8,9]. The disease results from a complex interaction between autoreactive immune cells, disruption of the blood–brain barrier, microglial activation, and loss of oligodendrocytes, leading to irreversible axonal damage [1,2,7,10].

Over the past two decades, the development of disease-modifying therapies (DMTs) has transformed the management of RRMS by reducing relapse rates and short-term inflammatory activity. However, these treatments remain less effective at preventing long-term disability accumulation and have a limited impact on neurodegeneration, which is the primary driver of irreversible clinical worsening. In progressive MS, inflammation becomes compartmentalized within the CNS, as blood–brain barrier permeability decreases and chronic neurodegeneration predominates, resulting in only limited benefit from current therapies. Moreover, high-efficacy DMTs are associated with significant risks, including infection, secondary autoimmunity, and malignancy, and none of the available agents reliably promote remyelination or neural repair [4]. This therapeutic gap highlights the urgent need for strategies that not only modulate immune activity but also target the degenerative and reparative dimensions of MS pathology.

These treatments aim to restore immune balance, reduce chronic inflammation, and support neural repair through trophic and regenerative mechanisms. Several cell types are under investigation, including autologous haematopoietic stem cells (AHSCT), which aim to reset the immune system after high-dose immunosuppression; mesenchymal stem cells (MSCs), which exert paracrine immunomodulatory and neuroprotective effects; and neural stem or progenitor cells (NSCs), which may contribute directly to remyelination and repair [11]. While many experimental and preclinical studies have shown promising results, clinical trials in humans are essential to determine safety, feasibility, and therapeutic impact. These studies provide the foundation for future translation into clinical practice.

This review examines the current clinical evidence on cell-based therapies for MS, describing their biological background, mechanisms of action, and therapeutic outcomes. It also discusses the main challenges that remain and highlights the priorities for future research needed to advance these therapies toward standard use in clinical care.

## 2. Methods

A systematic search was conducted in the databases PubMed, Scopus, and ClinicalTrials.gov to identify interventional clinical studies on cell therapy in human patients with multiple sclerosis (MS), published within the last five years. The review followed the PRISMA 2020 guidelines [12]; protocol was registered in web PROSPERO number, CRD420251236319. The search strategy, eligibility criteria, study selection provided, and data extraction procedures were predefined prior to initiating the review. Elegible studies were required to report clinical outcomes in MS patients undergoing any type of cell-based therapy, including hematopoietic stem cell transplantation (HSCT), mesenchymal stem cells (MSCs), adipose-derived stem cells, neural stem cells, oligodendrocyte precursor cells, and induced pluripotent stem cells. Only studies published in English were considered. Systematic reviews, meta-analyses, preclinical and in vitro studies, case reports without interventional treatment, and clinical trials without accessible outcome data were excluded.

An initial search was performed in July 2025. In PubMed, a combination of MeSH terms and free-text keywords related to MS and cell-based therapies was used. The final query included “multiple sclerosis” (MeSH or Title/Abstract) combined with relevant descriptors such as “cell therapy,” “stem cells,” “hematopoietic stem cells,” “non-hematopoietic stem cells,” “mesenchymal stem cells,” “adipose-derived stem cells,” “neural stem cells,” “oligodendrocyte precursor cells,” “oligodendrocytes,” “induced pluripotent stem cells,” and “iPSCs.” These terms were paired with clinical study identifiers (“clinical trial” as publication type or “clinical study” in Title/Abstract). Filters were applied to restrict results to studies involving humans, published in the previous five years, and categorized under clinical-trial–related article types. A total of 19 articles were retrieved. These records were manually reviewed, and studies not meeting the predefined eligibility criteria were excluded prior to screening.

The Scopus search was designed to maximize sensitivity by querying titles, abstracts, and keywords. The Boolean query combined “multiple sclerosis” with the same stem-cell–related terms used in PubMed. Additional filters restricted results to English-language publications, publication years > 2013, and article-type documents (DOCTYPE: ar). This search identified 714 documents. Titles and abstracts were exported to Excel for automated keyword filtering, generating 29 potentially eligible studies. These were subsequently examined manually to verify compliance with the inclusion criteria.

The ClinicalTrials.gov search used the terms “Multiple Sclerosis” combined with “Stem Cell,” “Cell Therapy,” “Hematopoietic Stem Cell,” “Mesenchymal Stem Cell,” “Neural Stem Cell,” and “Induced Pluripotent Stem Cell.” Filters were applied to include only interventional studies with posted results and with primary completion and first posted dates between 14 July 2020 and 14 July 2025. This search identified four completed trials with accessible results.

**Figure 1:**
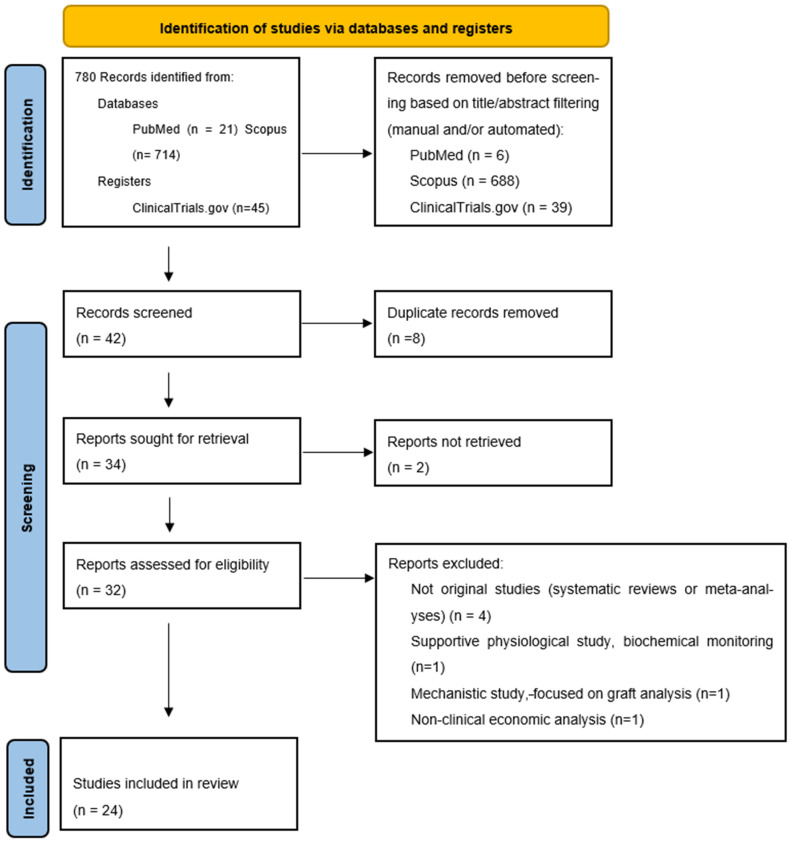
PRISMA 2020 flow diagram for study selection process.

All records retrieved from the three sources were compiled, and duplicates were removed. A total of 42 unique records were screened. After excluding eight duplicates, 34 studies remained for title and abstract screening. Full-text articles were sought for all 34 studies; however, two could not be accessed despite repeated attempts. Of the 32 studies undergoing full-text review, eight were excluded because they were systematic reviews, meta-analyses, or lacked clinical outcome data in human subjects. The final qualitative synthesis included 24 studies. The PRISMA flow diagram summarizing these steps is presented in Figure 1.

An additional search update was performed in September 2025 shortly before manuscript submission, identifying two recently published clinical trials indexed in PubMed. The PRISMA diagram was revised accordingly to reflect the inclusion of these two relevant studies.

Data extraction was performed using a predefined template. Extracted variables included authors and publication year, study phase and design, sample size, MS subtype, cell source and type, administered dose, route of administration, conditioning regimen when applicable, follow-up duration, safety outcomes, efficacy endpoints, and biological or imaging biomarkers. When studies included multiple treatment groups, doses, or administration schedules, all relevant outcomes were extracted. When information was unclear or incomplete, efforts were made to cross-check with Supplementary Materials or registry entries to confirm data. A formal risk-of-bias assessment was not conducted, consistent with the scoping nature of the review. However, study design characteristics (e.g., randomization, blinding, control group presence, sample size) were recorded and considered qualitatively when interpreting findings across heterogeneous cell-therapy approaches. Given the substantial variability in study designs, cell products, dosing regimens, routes of administration, outcome measures, and follow-up durations, quantitative synthesis or meta-analysis was not feasible. Therefore, a narrative synthesis was conducted, structured by cell-therapy type and integrating safety, efficacy, and biomarker findings across the included studies.

## 3. Biological Basis and Types of Cells Used

### 3.1. Immunological and Neuroregenerative Rationale

The rationale for cell-based therapies in MS is based on their dual capacity to modulate autoimmunity and promote repair within the CNS. MS is characterised by immune-mediated demyelination and neurodegeneration driven by autoreactive T and B lymphocytes, microglial activation, and impaired endogenous remyelination. Although DMTs effectively suppress inflammatory activity, none have achieved durable immune tolerance or reversed neuroaxonal loss. Cellular approaches aim to address these limitations through mechanisms of immune recalibration, neuroprotection, and tissue regeneration [13,14,15].

From an immunological perspective, cell therapies suppress autoreactive responses and restore immune balance by promoting regulatory populations such as Tregs and Bregs, downregulating proinflammatory cytokines, and modulating microglial activity. The clearest example is autologous haematopoietic stem cell transplantation (AHSCT), which combines immune ablation and reconstitution to achieve a functional “reset” of the immune repertoire [16]. Other cell types—such as mesenchymal stem cells (MSCs) and regulatory T cells (Tregs)—act primarily through paracrine immunomodulation, reducing chronic inflammation without destroying the immune system [17].

In parallel, several cell populations contribute to neurorepair. Neural stem cells (NSCs) and oligodendrocyte progenitor cells (OPCs) can differentiate into neurons and oligodendrocytes, facilitating remyelination and neuronal replacement [18,19,20,21,22,23]. MSCs also secrete neurotrophic and angiogenic factors—such as BDNF, GDNF, and VEGF—that promote axonal survival and synaptic recovery [24,25]. Paracrine mechanisms mediated by extracellular vesicles (EVs) further support remyelination, synaptic plasticity, and vascular stability [26,27,28,29,30]. Together, these mechanisms define the biological framework underpinning the therapeutic use of cells in MS: immune reprogramming coupled with repair of the damaged CNS environment.

### 3.2. Types of Cells Investigated in Humans

#### 3.2.1. Autologous Haematopoietic Stem Cell Transplantation (AHSCT)

AHSCT remains the most established cell-based intervention for MS, designed to eradicate autoreactive immune cells and rebuild a new, self-tolerant immune repertoire. The procedure involves three steps: mobilisation and collection of stem cells (usually from peripheral blood after cyclophosphamide and G-CSF administration), immunoablative conditioning, and reinfusion of the cryopreserved autologous product. Conditioning regimens vary in intensity, from reduced-dose cyclophosphamide with rabbit antithymocyte globulin (rATG) to higher-intensity BEAM-based protocols, chosen according to disease activity and patient risk profile [31,32,33].

The main toxicities derive from transient myelosuppression and infection risk during the aplastic phase. Long-term adverse effects may include infertility and secondary autoimmunity, though recent refinements have markedly reduced treatment-related mortality to below 1%. Logistically, AHSCT requires dedicated transplant facilities, close post-procedure monitoring, and strict infection control. Despite these complexities, it represents the most complete immune-reset strategy currently available [34].

#### 3.2.2. Mesenchymal Stem Cells (MSCs)

MSCs are multipotent stromal cells found in bone marrow, adipose tissue, umbilical cord, and placental tissue. They are isolated through density-gradient centrifugation and expanded in culture to yield a heterogeneous population capable of modulating immune and glial responses [35,36,37,38]. Their effects are largely mediated by secreted cytokines, growth factors, and EVs rather than by engraftment or differentiation [39].

Delivery routes for MSCs vary according to therapeutic intent. Intravenous (IV) administration offers systemic immunomodulation but is limited by poor homing of MSCs to the CNS and rapid clearance from circulation, reducing CNS bioavailability [40]. Intrathecal (IT) and intracerebroventricular (ICV) routes provide more direct CNS access but carry procedural risks such as headache or transient meningeal irritation [41,42,43]. Preclinical research has also explored intranasal delivery as a minimally invasive method capable of bypassing the blood–brain barrier and achieving CNS distribution without systemic exposure. While promising, this approach remains at an early experimental stage [44].

Major limitations of MSC therapy include donor variability, lack of standardisation in culture conditions, uncertain optimal dosing, and limited persistence after transplantation [45,46]. Despite these challenges, MSCs remain among the most of the most versatile and safe cell types under investigation for MS. Nevertheless, some studies in traumatic brain injury suggest an increased potential for tumorigenicity associated with their use [47].

#### 3.2.3. Neural Stem Cells and Oligodendrocyte Progenitor Cells (NSCs/OPCs)

NSCs and OPCs directly target the neuroregenerative aspects of MS by replacing or supporting damaged neural elements. NSCs can be derived from foetal tissue, adult brain niches, or induced pluripotent stem cells (iPSCs), whereas OPCs are usually generated from iPSCs or embryonic sources [48,49,50]. These cells have shown the ability to differentiate into myelin-producing oligodendrocytes, promote axonal remyelination and secrete neurotrophic factors that stabilise neural networks [49,51,52,53].

For therapeutic applications, administration is typically achieved via intrathecal or intracerebroventricular routes, ensuring CNS exposure but necessarily involving invasive procedures. Current barriers to clinical translation include low graft survival, limited migration towards demyelinated areas, and potential immunogenicity or tumourigenicity. The use of corticosteroids in MS may further inhibit OPC proliferation, representing a pharmacological challenge to successful engraftment [54]. Ongoing advances in cell engineering and biomaterial scaffolds aim to enhance the survival and integration of transplanted neural cells [11,55].

#### 3.2.4. Emerging Cellular Strategies

Novel cell-based and cell-free approaches are rapidly expanding the therapeutic landscape. Induced pluripotent stem cells (iPSCs) offer a renewable, autologous source for generating both immune and neural lineages, potentially overcoming donor variability and ethical constraints [56,57]. Extracellular vesicles (EVs) derived from MSCs or NSCs provide a cell-free approach for delivering microRNAs and proteins that regulate neuroinflammation and promote remyelination, with the advantage of easier storage, lower immunogenicity, and the ability to cross the blood–brain barrier [26,58,59]. Regulatory T-cell (Treg) therapy represents a targeted immunological strategy that restores immune tolerance through reinfusion of ex vivo–expanded autologous Tregs. Intrathecal administration is currently under investigation as a way to directly modulate CNS-compartmentalised inflammation [33,39]. Despite promising immunological effects, standardisation, scalability, and long-term persistence remain significant challenges across all emerging modalities.

#### 3.2.5. Summary and Comparative Analysis of Cell Therapies

**Figure 2:**
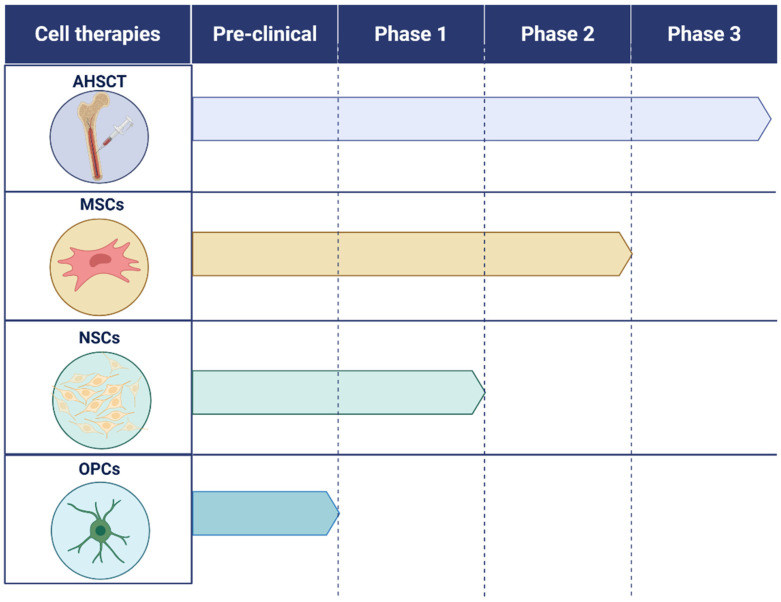
Clinical development status of the main cell-based therapies investigated for multiple sclerosis. The figure illustrates the progression through regulatory phases (preclinical, Phase 1, Phase 2, and Phase 3) of four therapeutic approaches: autologous hematopoietic stem cell transplantation (AHSCT), mesenchymal stem cells (MSCs), neural stem cells (NSCs), and oligodendrocyte progenitor cells (OPCs). The schematic compares the level of clinical maturity of each strategy, highlighting that AHSCT is the most advanced along the regulatory pathway, whereas NSCs and OPCs largely remain at early stages of research. Created in BioRender Pro. Gomez Pinedo, U. (2025) https://BioRender.com/ybp1w3p.

Although each cellular approach offers distinct therapeutic promise, their advantages and limitations differ substantially. AHSCT provides the most comprehensive and durable immune reconstitution and currently shows the strongest clinical efficacy in highly active RRMS; however, its use is limited by treatment-related toxicity, the need for specialized transplant facilities, and reduced benefit in progressive MS. MSCs represent a safer and more accessible option with broad immunomodulatory and neuroprotective properties, yet their clinical impact is modest, their CNS homing is inefficient, and cell products vary significantly between donors and manufacturing protocols. NSCs and OPCs directly target remyelination and neuroregeneration, offering theoretical advantages for progressive disease, but face major challenges including poor graft survival, invasive delivery procedures, and the risk of immunogenicity or tumorigenicity. Treg therapy provides highly targeted immune tolerance restoration but currently suffers from limited persistence, scalability issues, and manufacturing complexity. EV-based approaches circumvent many safety concerns associated with live-cell therapies and can cross the BBB, but remain in early development and lack evidence of durable clinical efficacy. Overall, no single cell strategy simultaneously fulfils all therapeutic goals—immune reset, inflammation control, neuroprotection, and remyelination (See Table 1) (Figure 2).

## 4. Clinical Evidence in Humans

Over the past two decades, cell-based therapies for MS have evolved from small safety trials to multicenter comparative studies. Early investigations primarily assessed feasibility and short-term tolerance in highly selected patients, whereas recent work has focused on randomised designs or registry-based evaluations comparing cellular approaches with high-efficacy DMTs. Collectively, these studies aim to determine whether immunological “resetting,” neuroprotection, and remyelination achieved through cellular interventions can translate into sustained subclinical benefit. The following subsections summarize the main clinical results obtained in human studies, organized according to the principal therapeutic strategy: (1) autologous hematopoietic stem-cell transplantation (AHSCT), (2) mesenchymal stem-cell (MSC)–based therapies, and (3) neural or other experimental cell approaches. Key outcomes and safety data are compiled in Table 2, Table 3 and Table 4.

### 4.1. Autologous Hematopoietic Stem Cell Transplantation (AHSCT)

AHSCT is a high-efficacy, single-intervention therapy designed to induce durable, drug-free remission in MS. The approach involves immune ablation through a conditioning regimen, followed by autologous stem cell reinfusion to reconstitute a naïve, self-tolerant immune repertoire. Over the past decade, the concept of immune “resetting” has been increasingly substantiated by strong evidence from randomized trials and extensive registry data.

#### 4.1.1. Clinical Efficacy Evidence

The strongest evidence for AHSCT efficacy derives from the Phase III MIST trial, which compared nonmyeloablative AHSCT (Cyclophosphamide 200 mg/kg + rATG 6 mg/kg) with continued disease-modifying therapy (DMT) in patients with highly active relapsing–remitting MS (RRMS). AHSCT was markedly superior in delaying disability progression (HR 0.07), with treated patients showing a mean improvement in EDSS (from 3.38 to 2.36), in contrast to worsening in the DMT arm (3.31 to 3.98) [60].

These findings have been corroborated by large-scale, real-world observational data. A 2025 comparative analysis by Kalincik et al. demonstrated that AHSCT outperformed other high-efficacy immune reconstitution therapies (Alemtuzumab, Ocrelizumab/Rituximab, Cladribine, Mitoxantrone) in relapse control, disability progression (CDA), and confirmed disability improvement (CDI) [61]. Similar registry-based studies found AHSCT to be superior to Fingolimod and Natalizumab, with efficacy comparable to Ocrelizumab at three years [62]. Moreover, Melnichenko et al. showed that even low-intensity conditioning regimens (e.g., reduced BEAM or Cy + Rituximab) can achieve durable disease control, with 83% relapse-free survival at seven years [63].

#### 4.1.2. Safety and Adverse Effects

Historically, treatment-related mortality (TRM) represented the principal limitation to AHSCT adoption. However, recent evidence shows a marked improvement in safety, driven by refined patient selection and reduced conditioning intensity. Both the MIST and MOST trials reported no TRM, while large registry analyses estimate modern TRM ≤ 1% (0.29–1.0%) in RRMS cohorts [60,61,62,64]. The 20-year UK registry by Kazmi et al. found all fatal cases occurred in patients with advanced disability (median EDSS 6.5), emphasizing the importance of appropriate candidate selection [65]. Further optimazation of conditioning regimens has reduced toxicity. Higher serotherapy doses (rATG ≥ 7.5 mg/kg) increased viral reactivation (notably CMV) without added efficacy, supporting lower-dose protocols (≤6 mg/kg). Post-discharge infections remain the leading cause of serious adverse events, accounting for nearly 60% in one registry [62]. Importantly, Kvistad et al. showed that prior exposure to high-efficacy DMTs does not increase transplant-related complications [66]. Melnichenko et al. reported zero TRM among 258 RRMS patients treated with low-intensity regimens, confirming the improved safety of contemporary AHSCT approaches [63] (Table 2).

#### 4.1.3. Limitations in Progressive Disease

While AHSCT has proven transformative for inflammatory RRMS, its benefit in progressive MS (SPMS/PPMS) remains limited. In a retrospective study Mariottini et al. compared AHSCT (BEAM + ATG) to low-dose immunosuppression in SPMS, finding complete suppression of relapse activity (100% RFS) but no significant advantage in preventing disability progression (P-FS) [67]. These findings reinforce the notion that AHSCT effectively eliminates the inflammatory component of MS but does not halt the non-inflammatory neurodegenerative processes that dominate the progressive phase.

#### 4.1.4. Summary and Recommendations

AHSCT is now recognized as the most efficacious therapy for RRMS, remaining unique in its capacity to induce disability improvement. Its safety profile has markedly improved in recent years, with treatment-related mortality consistently below 1% when performed in appropriately selected patients—those with active inflammatory disease and low-to-moderate disability. Reflecting this evidence, international guidelines from ECTRIMS and the EBMT now recommend AHSCT as a standard-of-care option for patients with highly active RRMS who have failed one or more high-efficacy disease-modifying therapies [68,69]. This shift represents a major paradigm change: from being considered a rescue intervention to a validated, frontline therapy in carefully selected cases. Given its complexity and potential risks, AHSCT should be performed in specialized centers following established guidelines. Further randomized Phase III trials and long-term follow-up studies are needed to confirm its durability and clarify the mechanisms underlying sustained remission (Table 2).

### 4.2. Mesenchymal Stem Cell (MSC) Therapies

MSC therapies are being explored as an alternative to immune ablation, focusing on immunomodulation, neuroprotection, and potential myelin repair. In contrast to AHSCT, MSCs are not intended to reconstitute the immune system but to modulate it and provide trophic and anti-inflammatory support within the CNS. Over recent years clinical evidence remains heterogeneous, with efficacy outcomes influenced by the cell source, route of administration, and disease phenotype.

#### 4.2.1. Evidence from Intravenous (IV) Administration

The intravenous (IV) route has been the most frequently investigated in patients with inflammatory relapsing-remitting MS (RRMS) and active progressive MS, though with variable efficacy.

The largest randomised controlled trial to date (RCT), MESEMS, enrolled 144 participants with active RRMS or progressive MS [70]. It evaluated a single IV infusion of autologous bone marrow-derived MSCs (BM-MSCs) but did not meet its primary endpoint of reducing cumulative new gadolinium-enhancing (GdE) MRI lesions. A neurophysiological sub-study, MESCAMS, confirmed the absence of a measurable impact on inflammatory or functional outcomes [71].

By contrast, other studies using repeated dosing or alternative MSC sources have produced more encouraging findings. The HBMS01 Phase II RCT investigated multiple IV infusions of autologous adipose-derived MSCs (HB-adMSCs) in 24 RRMS patients [72]. The study achieved its primary endpoint, demonstrating statistically significant improvements in EDSS, quality-of-life (MSQoL-54) and mood scores compared with placebo. Similarly, a small Phase I trial of allogeneic placenta-derived MSCs (PL-MSCs) in five patients with secondary progressive MS (SPMS) showed good tolerability and exploratory clinical benefits, including reductions in EDSS and fatigue measures [73] (Table 3).

#### 4.2.2. Evidence from Intrathecal (IT) and Combined Routes in Progressive MS

To improve CNS targeting and address neurodegenerative mechanisms, several studies have focused on intrathecal (IT) or combined IT + IV administration, mainly in progressive MS (PMS).

The MSC-NP programme, using autologous BM-derived neural progenitor-like MSCs, established long-term safety in a Phase I study [74]. In its subsequent Phase II RCT involving 54 PMS participants, the therapy was well tolerated but did not achieve its primary endpoint (EDSS-Plus). Nonetheless, secondary analyses revealed a significant reduction in grey-matter atrophy, suggesting a potential neuroprotective effect [43].

The strongest evidence for neuroprotection in PMS arises from biomarker studies. Petrou et al., analysing cerebrospinal fluid (CSF) from a Phase II RCT (*n* = 48, PMS), demonstrated that IT-MSC therapy significantly reduced CSF markers of neurodegeneration (neurofilament light chain, NFL) and compartmentalised inflammation (CXCL13) compared with placebo, providing biological proof-of-concept [75]. This was reinforced in the MSC-NG01 open-label extension [17], where repeated IT MSC injections (N = 23, PMS) resulted in sustained reductions in serum biomarkers of neurodegeneration (NFL—32%) and astroglial activation (GFAP—23.2%) over one year, correlating with improvements in cognition and walking ability [76]. Similarly, the NurOwn Phase II trial, using IT MSCs induced to secrete neurotrophic factors (MSC-NTF), confirmed safety and showed favourable modulation of neuroprotective CSF biomarkers [77] (Table 3).

Studies employing combined IT + IV routes further support these findings. Pluchino et al. [22] reported that repeated IT + IV autologous BM-MSC administrations in 24 PMS patients resulted in clinical stability or improvement in 22 participants during extended follow-up [78]. A Phase I/II dose-finding study of allogeneic umbilical cord-derived MSCs (UC-MSCs) in RRMS, SPMS, and PPMS populations also demonstrated dose-dependent improvements in clinical and cognitive measures with preservation of grey-matter volume [79].

#### 4.2.3. Safety and Adverse Events

Across all recent trials, MSC therapy has demonstrated a favourable safety profile irrespective of cell origin (autologous bone marrow, adipose, placental, or umbilical) or route of delivery. Immunogenicity has not emerged as a major concern, and adverse events are generally mild and transient—typically headache, low-grade fever and back pain—often related to the IT procedure rather than to the cell product itself.

#### 4.2.4. Summary

Overall, the clinical evidence for MSC therapy in MS indicates that its therapeutic effects are highly context-dependent. The negative outcome of the MESEMS trial suggests that single IV doses are insufficient to control acute inflammatory activity in active MS. In contrast, studies in progressive MS using IT or combined IT + IV administration have shown consistent biological efficacy, evidenced by reductions in neurodegeneration (NFL, GFAP) and inflammatory (CXCL13) biomarkers, as well as by MRI evidence of tissue preservation. Although these biological effects have not yet translated into statistically significant improvements in primary clinical endpoints, they provide a strong rationale for further investigation of MSCs as a neuroprotective and disease-modifying approach in progressive forms of MS.

**Table 2:** Summary of Clinical Studies on AHSCT in MS (2020–2025).

Abbreviated Study Name (Ref.)	Publication Year	Study Design	Sample (*n*)	Conditioning Regime	MS Type/Population	Main Outcomes/Safety
MOST (NCT03342638) [64]	2020	Phase III, randomized, open-label, 2-arm (Cyclophosphamide + rATG ± IVIg; USA; 2017–2019)	*n* = 66	Intermediate (Lymphoablative): Cyclophosphamide + rATG + Methylprednisolone	RRMS, EDSS 2.0–6.0, refractory to ≥1 DMT	Primary endpoint (5-yr NEDA) not collected (trial terminated early). No TRM; no SAEs within 1-yr FU.
MIST (NCT00273364) [60]	2020	Phase III, randomized, controlled, multicenter (2005–2018; USA, Sweden, UK, Brazil; FU 5 years)	*n* = 110	Low/Intermediate (Nonmyeloablative): Cyclophosphamide (200 mg/kg) + rATG (6 mg/kg)	RRMS, 18–55 yrs, EDSS 2.0–6.0, ≥2 relapses or MRI activity despite DMT	HSCT delayed progression vs. DMT (HR 0.07, *p* < 0.001); EDSS 3.38→2.36 vs. 3.31→3.98; NEDA 98.1% (1 yr) vs. 20.8%. TRM 0%; no grade 4 toxicities.
TCR Repertoire Reconstitution [80]	2020	Translational, prospective comparative vs. Natalizumab (24-month FU; Italy)	*n* = 15	Intermediate/High (Myeloablative): BEAM protocol + rATG (7.5 mg/kg)	RRMS	AHSCT: complete TCR β reconstitution; ↑ diversity vs. NTZ; indicates deeper immune reset.
AHSCT with Low-Intensity Conditioning [63]	2021	Observational, prospective single-center (2006–2018; Russia; median FU 30 months, up to 7 years)	*n* = 258	Low-intensity: BEAM-like ± ATG or Cyclophosphamide (200 mg/kg) + Rituximab	RRMS	99% clinical response; EDSS 2.0→1.5 stable >5 yrs; QoL improved; RFS 83%, PFS 86% (7 yrs); no RFS diff. between regimens. No TRM; No difference between regimes: grade 2–3 hepatic toxicity 20.5%; mucositis 1.6%; transient neurologic worsening 6.4%; neutropenic fever 27%; anemia (grade 3) 1.9% allergic reactions, 2.3%.
Impact of Previous DMT [66]	2022	Observational, retrospective multicenter (2011–2021; Sweden & Norway)	*n* = 104	Intermediate (Lymphoablative/Nonmyeloablative): Cyclophosphamide (200 mg/kg) + rATG (6 mg/kg)	RRMS	Mean FU 39.5 months; 81% NEDA-3; prior long-acting DMTs sustained 100% NEDA-3 (*p* < 0.01); efficacy/toxicity unaffected by prior DMT. No TRM; neutropenic fever ~66%; secondary autoimmunity ~19% (thyroid); no significant group differences.
AHSCT vs. Low-Dose Immunosuppression in SPMS [67]	2022	Retrospective, monocentric matched-cohort (mean FU 91–99 months; Italy)	*n* = 93	Intermediate (Myeloablative): BEAM protocol + ATG vs. low-dose Cyclophosphamide	SPMS	AHSCT suppressed relapses (R-FS 100% vs. 52%, *p* < 0.0001); P-FS similar (70% vs. 81%); no NEDA difference. No AHSCT deaths; 2 deaths (Cyclophosphamide group); 3 malignancies (1 AHSCT, 2 Cyclophosphamide).
AHSCT vs. Fingolimod, Natalizumab, Ocrelizumab [62]	2023	Observational, multicenter, registry-based, propensity-matched (2006–2021; Canada, Sweden, UK, Norway, Australia)	*n* = 4915	Variable Intensity (Mixed): Regimens included BEAM, Busulfan + Cyclophosphamide, or Cyclophosphamide (200 mg/kg) + ATG. 84% received ATG	RRMS (highly active)	AHSCT superior to Fingolimod and Natalizumab (ARR 0.09 vs. 0.20/0.10; HR ~2.7); similar efficacy to Ocrelizumab. 1 TRM; early complications: neutropenia 23%, serum sickness 11%, ICU 8.8%; 82 SAEs after discharge (59.8% infections, mostly viral).
HSCT vs. IRT in RRMS [61]	2025	Observational, registry-based, multicenter (2006–2023; 32 countries, 117 centers)	*n* = 3901	Variable Intensity (Mixed): 55% Low (lymphoablative), 25% Intermediate (myeloablative), 20% High. Regimens primarily Cyclophosphamide + ATG, BEAM, or Busulfan + Cyclophosphamide	RRMS	AHSCT superior to all comparators (high-efficacy immune reconstitution therapies: Alemtuzumab, Ocrelizumab/Rituximab, Cladribine, Mitoxantrone for relapse control (HR 0.35–0.53), disability improvement (CDI), and progression slowing (CDA). 1-year TRM 0.29%.
AHSCT in the UK (BSBMTCT) [65]	2025	Retrospective, 20-year registry-based cohort (2002–2023; UK)	*n* = 364	Low/Intermediate Intensity: Predominantly Cyclophosphamide (200 mg/kg) + rATG (rabbit ATG). The rATG dose varied (6.0 mg/kg or 7.5 mg/kg), with 98% of patients receiving this Cy/rATG regimen. A small minority (2%−3%) received BEAM/ATG.	RRMS (58%) and progressive MS (36%)	5-year PFS 62.4%; significantly higher in RRMS than progressive MS. 100-day TRM 1.4%; occurred only in patients with baseline EDSS ≥ 6.5. rATG doses ≥ 7.5 mg/kg (used in 61% of patients) were associated with increased viral reactivation (CMV) and conferred no superior clinical benefit compared to rATG ≤ 6.0 mg/kg.

Arrows: ↑ = increase.

**Table 3:** Summary of Clinical Studies on MSC in MS (2020–2025).

Abbreviated Study Name (Ref.)	Publication Year	Study Design	Sample (*n*)	MSC Source and Administration Route	MS Type/Population	Main Outcomes	Adverse Events
MESEMS [70]	2021	Phase II RCT, double-blind, placebo-controlled, crossover; 2014–2017 (9 European countries + Canada)	144	Autologous BM-MSCs (IV, 1–2 × 10^6^/kg)	Active RRMS/Progressive MS, 18–50 y, ≥1 DMT failure	No ↓ in GELs (RR 0.94; *p* = 0.78); no effect on ARR, EDSS, MSFC, SDMT. Safe but not efficacious.	AE rates balanced; 25% infection; no treatment-related SAEs.
MSC-NP (Phase I) [74]	2021	Open-label, 1 arm; 2014–2018 (USA)	20	Autologous MSC-NPs (IT, 3 injections)	Progressive MS	Safe at 2 yrs; MRI stable; 7/20 EDSS improved but not maintained in 5 cases ↓ CCL2, ↑ HGF, IL-8, CXCL12.	No treatment-related SAEs.
Petrou et al. (Long-term Follow-up) [78]	2021	Open prospective extension: recruitment not specified (Israel)	24	Autologous BM-MSCs (IT + IV)	Active Progressive MS, 18–50 y, refractory to approved therapies	EDSS stable/improved in 22/24 (6.75 → 6.42; *p* = 0.028); relapses ↓ (16 → 3; *p* = 0.002). >2 injections → better response.	No SAEs; mild HA/fever (IT-related).
MESCAMS [71]	2022	RCT, double-blind, sham-controlled, crossover. 2014 –2017 (Canada)	20	Autologous BM-MSCs (IV infusion)	RRMS/Progressive MS	No clinical benefit; ↑ MEP latency & CMCT indicating motor decline.	Not described.
Petrou et al. (Biomarker Study) [75]	2022	Phase II RCT, double-blind, placebo-controlled; 2015–2019 (Israel)	48	Autologous BM-MSCs (IT or IV)	Progressive MS	CSF NF-L ↓ (*p* = 0.026); CXCL13 ↓ vs. ↑ in placebo (*p* = 0.012). no definite statistical correlation between clinical parameters and NF-L levels,	Not described.
NurOwn (BCT-101) [77]	2023	Phase II open-label, 1 arm. 2019 –2021 (USA)	18	Autologous MSC-NTF (BM-derived, IT)	Progressive MS EDSS 3.0–6.5	19% ≥ 25% improvement in T25FW/9HPT; ↑ neuroprotective and ↓ inflammatory cytokines.	No TRM. Post-injection pain, arachnoiditis; no SAEs.
HBMS01 [72]	2024	Phase II RCT, double-blind, placebo-controlled; 2021–2024 (USA)	24	Autologous adipose-derived MSCs (IV, 6 infusions)	RRMS, EDSS 3.0–6.5, stable on DMT	↑ MSQoL-54 Physical (+22.1) & Mental (+15.8); EDSS ↓ 1.55; ↓ depression (*p* < 0.05).	No deaths; 1 SAE/arm; headache common (67%).
MSC-NP (Phase II) [43]	2024	Phase II RCT, double-blind, placebo-controlled; 2018–2022 (USA)	54	Autologous MSC-NPs (IT, 6 inj/2 mo)	Progressive MS, non-active MRI	Primary endpoint not met; EDSS 6–6.5 subgroup ↑T25FW/6MWT (*p* = 0.03); ↓ GM atrophy ↑MMP9, ↓ CCL2.	No TRM; HA common (34% vs. 15%).
Umbilical Cord-MSCs (Phase I/II) [79]	2024	Phase I/II open-label, comparative; 2021 –2023 (Jordan)	35 (20 A, 15 B)	Allogeneic Umbilical Cord -MSCs + secretome (IT + IV)	RRMS/Progressive MS	Safe; 2- dose (A) > 1-dose (B): ↑ EDSS, SDMT, BDI, 9HPT; ↓ lesion burden; preserved GM volume ↑ CD4^+^ T, ↓ TNFα. TAP-1, miR142.	No SAEs: mild HA/fever, lumbar pain resolved.
MSC-NG01 (Extension) [76]	2025	Open-label extension; 2018–2023 (Israel)	23	Autologous MSC-NG01 (IT, 2–3 inj/6 mo)	Progressive MS	CSF NFL ↓ 32% (*p* = 0.001); GFAP ↓ 23%; ↑ T25FW (+4.5%), ↑ SDMT (+3 pts): ↑ QoL/fatigue.	No SAEs; transient HA/back pain.
Placenta-MSCs (Phase I) [73]	2025	Phase I open-label safety trial; 2022–2024 (Iran)	5	Allogeneic placenta derived MSCs (IV)	SPMS refractory to DMTs	Safe; EDSS ↓ (*p* < 0.0001); ↑cognitive & psychological scores; ↓ fatigue/anxiety.	Mild HA (2/5) resolved

Arrows: ↑ = increase, ↓ = decrease.

**Table 4:** Neural, Bone Marrow–Derived, and Other Cell-Based Therapies in Multiple Sclerosis.

Study (Ref.)	Publication Year	Study Design	Sample (*n*)	Cell Type/Source	MS Type/Population	Main Outcomes	Adverse Events
Treg Study [81]	2021	Phase Ib/IIa, open-label, two-arm (IV vs. IT) (2015–2019; Poland)	14 (11 IV, 3 IT)	Autologous ex vivo expanded Tregs (CD4^+^CD2^high^CD127^−^)	Relapsing-Remitting MS (RRMS)	Safe and well-tolerated. IT route demonstrated superior efficacy: IT group (0 relapses, stable EDSS/MRI) vs. IV group (12 relapses, ↑ T2 lesions).	No SAEs reported.
SIAMMS II [82]	2022	Phase I extension, prospective, open-label (2018–2020; UK)	4	Autologous, unfractionated bone marrow cells (BMCs), Intravenous (IV)	Progressive MS (SPMS/PPMS)	Repeat infusion was safe and feasible. Clinical and MRI measures were stable. Limited efficacy signal (attributed to low dose & IV route).	No SAEs. Mild, procedure-related AEs (pain, rash).
STEMS [83]	2023	Phase I, open-label, non-randomized (2017–2020; Italy)	12	Human fetal neural precursor cells (hfNPCs), Intrathecal (IT)	Progressive MS (PMS); EDSS ≥ 6.5	Feasible, safe, and tolerable. Primary safety outcome met. Exploratory: correlation between high-dose and reduced gray matter atrophy rate.	No severe adverse reactions related to hfNPCs.
hNSC-SPMS [84]	2023	Phase I, open-label, dose-escalation (2017–2020; Italy)	15	Allogeneic human neural stem cells (hNSCs), Intracerebroventricular (ICVI)	SPMS; Mean EDSS 7.6	Feasible, safe, and tolerable. Clinical & MRI stability. Exploratory: inverse correlation between NSC dose and parenchymal brain volume changes.	No treatment-related deaths or SAEs. Mild, transient procedure-related AEs (e.g., headache).

AHSCT (Autologous Hematopoietic Stem Cell Transplantation), ARR (Annualized Relapse Rate), ATG (Anti-Thymocyte Globulin), BEAM (Carmustine, Etoposide, Cytarabine, Melphalan), BSBMTCT (British Society of Blood and Marrow Transplantation and Cellular Therapy), CD (Confirmed Disability), CD34+ (Hematopoietic stem cell marker), CDA (Confirmed Disability Accumulation), CDI (Confirmed Disability Improvement), DMT (Disease-Modifying Therapy), EDSS (Expanded Disability Status Scale), FU (Follow-Up), G-CSF (Granulocyte-Colony Stimulating Factor), HR (Hazard Ratio), HSC (Hematopoietic Stem Cell), HSCT (Hematopoietic Stem Cell Transplantation), ICU (Intensive Care Unit), IRT (Immune Reconstitution Therapy), IV (Intravenous), IVIg (Intravenous Immunoglobulin), MIST (Multiple Sclerosis International Stem Cell Transplant), MOST (Maximizing Outcome of Multiple Sclerosis Transplantation), NEDA (No Evidence of Disease Activity), PFS (Progression-Free Survival), rATG (rabbit Anti-Thymocyte Globulin), RFS (Relapse-Free Survival), RRMS (Relapsing-Remitting Multiple Sclerosis), SAE (Serious Adverse Event), SPMS (Secondary Progressive Multiple Sclerosis), TCR (T-Cell Receptor), TRM (Treatment-Related Mortality). Arrows: ↑ = increase, ↓ = decrease.

### 4.3. Neural, Bone Marrow–Derived, and Other Emerging Cell Therapies

Beyond AHSCT and MSC-based strategies, several emerging cellular approaches aim to achieve either direct neuroprotection or targeted immune modulation. Current evidence—summarised in Table 4—is restricted to early-phase studies primarily assessing safety and feasibility.

Two Phase I trials have explored the direct administration of neural-lineage cells into the CNS of patients with progressive MS. The STEMS trial, an open-label study in 12 participants, found that intrathecal (IT) delivery of human fetal neural precursor cells (hfNPCs) was well tolerated, with exploratory analyses indicating a dose-dependent reduction in grey matter atrophy [70]. Similarly, the hNSC-SPMS trial (*n* = 15) tested intracerebroventricular (ICV) infusion of allogeneic human neural stem cells (hNSCs) [83]. This dose-escalation study confirmed procedural feasibility and reported clinical and MRI stability, with an inverse correlation between administered cell dose and parenchymal brain volume loss. Collectively, these findings support the safety of direct CNS administration and suggest potential neuroprotective activity. Other early approaches have investigated alternative cell types. The SIAMMS II study, a Phase I extension, evaluated repeated intravenous (IV) infusions of autologous unfractionated bone marrow cells in progressive MS [81]. Although the treatment was well tolerated, no clear clinical or radiological benefit was observed, likely reflecting limited CNS delivery due to pulmonary cell sequestration following IV infusion. A Phase Ib/IIa trial of autologous regulatory T cells (Tregs) in relapsing-remitting MS [19] further emphasised the relevance of administration route. Both IV and IT delivery proved safe, yet the IT arm (*n* = 3) showed superior outcomes, with no relapses or MRI progression, compared with continued disease activity in the IV arm (*n* = 11).

Taken together, these studies indicate that reparative (NSC) and precision immunomodulatory (Treg) therapies are biologically feasible and safe. Their clinical potential, however, seems to depend critically on direct CNS administration to bypass the blood–brain barrier and enhance local therapeutic engagement.

### 4.4. Synthesis and Comparative Evidence

The clinical evidence presented in this section illustrates the diversity of cellular strategies under investigation for MS, ranging from complete immune system reconstitution to targeted neuroprotection. AHSCT remains the most established and effective cellular therapy, supported by high-level evidence from randomised controlled trials and extensive registry analyses. It provides a durable “immune reset” capable of long-term remission and confirmed disability improvement in patients with highly active RRMS. Comparative data consistently demonstrate superior efficacy over high-efficacy disease-modifying therapies (DMTs), with a treatment-related mortality (TRM) below 1% when modern intermediate-intensity conditioning protocols and rigorous patient selection are applied. In contrast, MSC therapies show an excellent safety profile but variable efficacy across studies. A clear pattern emerges based on administration route: single intravenous (IV) infusions, as used in the MESEMS trial, failed to control inflammatory MRI activity, whereas intrathecal (IT) or combined IT + IV administration in progressive MS (PMS) has shown consistent biological effects. These include reductions in biomarkers of neurodegeneration (neurofilament light chain—NFL) and astroglial activation (GFAP), alongside evidence of grey-matter preservation. Although these biomarker and imaging outcomes have not yet translated into robust clinical improvement, they provide strong mechanistic support for MSCs as neuroprotective and potentially disease-modifying agents in PMS. NSC and Treg therapies represent the next generation of precision approaches, targeting either direct CNS repair or highly specific immune tolerance. Early-phase studies confirm their safety and feasibility, with exploratory results suggesting that therapeutic efficacy depends on direct CNS administration—such as intrathecal (IT) or intracerebroventricular (ICV) routes—to bypass the blood–brain barrier and achieve local immunomodulation and neuroregeneration.

## 5. Limitations and Challenges

Despite growing clinical interest, the clinical translation of cell-based therapies for MS face different challenges by several biological, methodological regulatory and economic limitations.

A fundamental limitation arises from the marked heterogeneity of MS pathology, ranging from RRMMS to PMS with different levels of baseline disability and prior exposure to disease-modifying therapies. Each patient has a variable degree of inflammation, demyelination, and neurodegeneration that complicates the selection of an appropriate cell type and the optimal timing for intervention. RRMS is dominated by inflammation and appears more responsive to immunomodulatory interventions, whereas PMS is characterized by neurodegeneration, glial scarring, reduced oligodendroglial recruitment, and a hostile and inhibitory microenvironment that limits the efficacy of transplanted cells to promote repair or remyelination [85].

**Figure 3:**
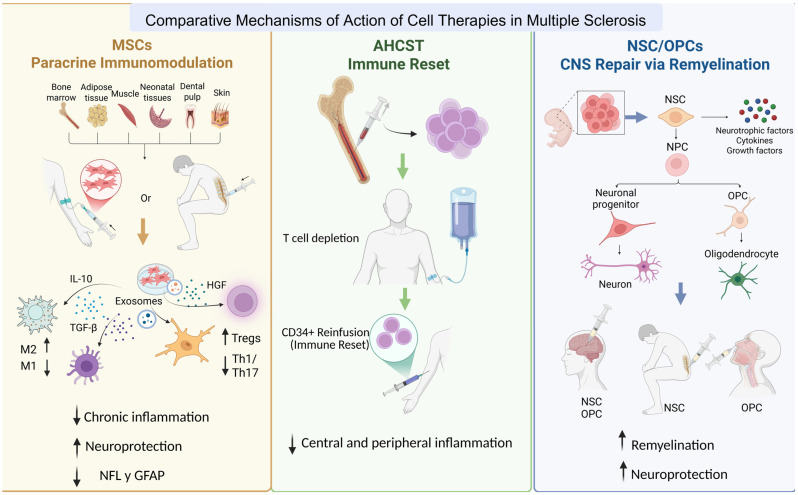
Comparative mechanisms of action of cell-based therapies applied to multiple sclerosis. The figure summarizes the key biological mechanisms associated with each cellular approach. MSCs primarily act through paracrine immunomodulation, reducing chronic inflammation. AHSCT induces an “immune reset” via immune ablation followed by reinfusion of CD34^+^ cells, thereby decreasing both central and peripheral inflammation. In contrast, NSCs and OPCs contribute to central nervous system repair by providing trophic factors, generating neuronal and glial progenitors, and promoting remyelination and neuroprotection. (Arrows: ↑ = increase, ↓ = decrease). Created in BioRender. Gomez Pinedo, U. (2025) https://BioRender.com/5gmvheo.

A second major challenge involves the lack of standardisation in cell sourcing, manufacturing, dosing, and delivery. Studies differ widely in cell origin, expansion protocols, and quality-control criteria, resulting in products that are not biologically equivalent. Delivery methods (intravenous, intrathecal, intracerebral) differ substantially in both efficacy and safety. In general, the more invasive the route of administration, the greater the procedural risks. The challenge is not only that studies use different routes, but that the route itself creates a biological limitation: cell-intrinsic properties determine which delivery methods are feasible or effective. For example, AHSCT and regulatory T cells are administered intravenously because they act primarily through systemic immune reconstitution or modulation rather than direct CNS integration. MSCs can also be delivered intravenously, but they rarely reach the CNS in significant numbers due to first-pass entrapment in peripheral organs. For this reason, intrathecal administration is often explored to enhance their CNS bioavailability. In contrast, more fragile or lineage-committed cells—such as NPCs or OPCs—show limited migration capacity and reduced survival after systemic delivery. These cell types typically require intrathecal or even intracerebroventricular administration to achieve adequate homing, survival, and integration within the CNS. These invasive routes require specialised neurosurgical expertise and dedicated clinical facilities, adding logistical complexity and further constraining their widespread clinical application. The ideal dosing regimen is not established and protocols differ significantly across studies. The durability of therapeutic effects is also uncertain. MSC-based effects are predominantly mediated through paracrine mechanisms, and infused cells have limited survival within the CNS. Many early-phase trials report short-term immunological or biomarker improvements followed by waning effects over months, suggesting that dosing intervals and long-term schedules have not been optimized [32,46] (Figure 3).

Methodological limitations further complicate progress. One major issue is patient selection with different disease subtypes and stages, and mixing these phenotypes in early-phase studies makes it difficult to interpret therapeutic effects. Equally important is the absence of robust, validated biomarkers capable of reliably measuring remyelination, neuroprotection, or long-term repair. Current trials often rely on clinical endpoints—such as EDSS or relapse rate—that are insensitive to subtle repair processes and may reflect transient immunomodulation rather than genuine tissue restoration. This mismatch between biological targets and outcome measures complicates efficacy evaluation.

All of the variations explained above complicate cross-trial comparisons, contribute partially to the inconsistent clinical outcomes, reduce the ability to identify which subgroups benefit the most from each cell type and slows the translation of cell-based therapies into clinical practice [46,70].

Long-term safety remains insufficiently defined. Most cell-based therapies beyond AHSCT have been evaluated only in small cohorts with limited follow-up, restricting the ability to detect delayed adverse events or sustained benefit [33,86]. Although short-term safety profiles are generally favorable, several potential long-term risks remain a concern, including immune sensitization to allogeneic cell products, immune rejection, infection, ectopic tissue formation, and tumorigenicity, particularly when pluripotent or embryonic-derived cells are used. In the case of AHSCT, treatment-related mortality has markedly declined in recent years, but gonadotoxicity from conditioning regimens—and the resulting infertility risk—remains a clinically significant issue, and must be carefully weighed, especially for younger patients.

Economic and regulatory barriers add further complexity. Cell therapies involve high upfront costs, and comprehensive cost-effectiveness data remain limited. However, a nationwide Polish study assessing AHSCT demonstrated an 82% reduction in overall treatment costs and a 97% reduction in DMD expenditures within one year post-transplant, suggesting potential long-term economic benefit. Such economic evaluations remain scarce and context-specific, emphasizing the need for broader health-economic assessments to inform sustainable clinical implementation [87]. Regulatory frameworks also hinder progress: no cell-based therapy is currently approved for MS by agencies such as the FDA or EMA. As a result, all approaches remain investigational, and there is no unified regulatory or manufacturing standard governing product characterization, dosing, or delivery. This lack of formal approval contributes to the substantial heterogeneity seen across studies and complicates direct comparisons of efficacy and safety [51,70].

Ethical considerations vary widely across cell types. Autologous products such as AHSCT, MSCs or Tregs pose minimal ethical concerns, whereas neural stem cells and OPCs derived from fetal tissue raise substantial ethical and regulatory challenges, including complex consent procedures and stringent oversight requirements.

Altogether, these challenges stress why the clinical translation of cell-based therapies for MS remains limited and why current evidence is insufficient to support widespread implementation. Progress in this field will require greater standardisation of manufacturing and delivery methods, clearer patient-selection strategies, validated biomarkers capable of capturing repair, and robust long-term safety data. Strengthening these areas will be essential to define the true therapeutic potential of these approaches and support their responsible integration into future MS care.

## 6. Future Perspectives

Future progress in cell-based therapies for MS will require coordinated advances across several key areas. Refining the selection and engineering of therapeutic cell types remains essential. Ongoing work with autologous MSCs, neural precursor cells, and regulatory T cells aims to enhance immunomodulatory and reparative properties while reducing immunogenicity and tumourigenic risk. Genetically engineered products—such as CAR-Treg cells or stem cells modified to enhance trophic factor secretion—offer promising opportunities for more targeted and durable effects [88].

Improving delivery methods is another major priority. Current routes differ in feasibility, invasiveness, and CNS penetration. Approaches such as cell pre-conditioning, biomaterial scaffolds, and strategic integration with existing disease-modifying therapies (DMTs) may enhance cell survival, improve CNS homing, and create a more permissive environment for repair. Sequential or combined strategies could reduce inflammatory activity before cell administration or sustain immunoregulatory effects afterwards, thereby maximising clinical benefit. The intranasal route is also attracting increasing interest because it offers minimally invasive access to the CNS and could ultimately support self-administration, improving adherence and reducing healthcare burden [89]. Early human data, including intranasal MSC delivery in neonates, demonstrate feasibility and justify further optimisation of formulations, dosing, and delivery devices [90].

Enhancing the precision and functional performance of therapeutic products will also be crucial. Gene-edited regulatory cells, iPSC-derived lineages, and cell-free strategies such as extracellular vesicles may help overcome limitations associated with live-cell transplantation while preserving key paracrine mechanisms. These innovations have the potential to improve safety, scalability, and consistency across clinical applications [91].

Progress will further depend on the development of sensitive and reliable biomarkers. Advances in single-cell omics, molecular imaging, and electrophysiological tools are generating more accurate indicators of remyelination, axonal integrity, and immune modulation. Validated biomarkers will be essential for patient stratification, early assessment of therapeutic effects, and regulatory approval [37,92].

Standardisation and regulatory harmonisation remain central to successful translation. Unified protocols for manufacturing, potency testing, and quality control will enhance reproducibility across centres. Regulatory frameworks also require alignment, as classification differences across jurisdictions complicate multicentre trial design. Clinical experience has already shown that improved patient selection—particularly in AHSCT—has markedly reduced treatment-related mortality, highlighting the importance of refined criteria that prioritise younger patients with aggressive RRMS and preserved organ function.

Economic and practical considerations will continue to influence clinical adoption. Although early data suggest that some cell-based approaches may reduce long-term healthcare costs, robust health-economic analyses across diverse settings are needed. Non-invasive delivery routes and simplified manufacturing platforms may further increase accessibility and sustainability.

Overall, the convergence of stem-cell engineering, biomaterials, gene editing, and advanced diagnostics is expected to shape the next generation of cell-based and cell-derived therapies for MS. Continued progress in manufacturing, delivery, biomarker development, and regulatory alignment will be essential to realise their therapeutic potential and facilitate their responsible integration into future MS care.

## 7. Conclusions

Cell-based therapies are a promising addition to the therapeutic landscape of multiple sclerosis, with potential to address both immune dysregulation and neurodegeneration. Among current approaches, autologous haematopoietic stem cell transplantation (AHSCT) has the strongest evidence for sustained remission in highly active relapsing disease, whereas mesenchymal and neural stem cell strategies offer possibilities for neuroprotection and repair, although their clinical efficacy remains preliminary.

Despite encouraging safety profiles and consistent biological activity, most cell-based interventions remain investigational. Variability in patient selection, manufacturing protocols, delivery routes and outcome measures limit comparability across studies and contribute to inconsistent results. Long-term safety—particularly for pluripotent or lineage-committed products—also requires more robust evaluation.

Future progress will depend on methodological harmonisation, improved patient stratification, optimization of dosing, development of minimally invasive delivery routes, and reliable biomarkers capable of capturing remyelination and neuroprotection. Integration of cell-based therapies with existing disease-modifying treatments may further enhance outcomes by combining immunological control with regenerative support.

Overall, although challenges remain, advances in stem-cell engineering, manufacturing standards, imaging, and regulatory frameworks support cautious optimism. With continued rigorous investigation, cell-based therapies have the potential to evolve from experimental interventions to clinically meaningful options for selected patients with MS.

## Figures and Tables

**Table 1:** Comparative Table of Cell-Based Therapies in MS.

Cell Type	Source	Mechanism of Action	Administration Route	Clinical Stage	Advantages	Limitations
Autologous Hematopoietic Stem Cell Transplantation (AHSCT)	Autologous peripheral blood or bone marrow	Immune ablation and immune reconstitution; elimination of autoreactive lymphocytes	Intravenous after conditioning	Phase II/III trials	Most potent immune reconstitution Strongest clinical evidence Effective in aggressive RRMS Single-procedure therapy	Requires immunoablation (toxicity) Infertility, autoimmunity, infection risk, TRM Limited benefit in progressive MS Requires transplant centers
Mesenchymal Stem Cells (MSCs)	Bone marrow, adipose tissue, umbilical cord, amniotic tissue	Immunomodulation, neuroprotection, trophic support, promotion of endogenous repair	Intravenous, intrathecal, intranasal	Phase I/II trials; safety established, efficacy under investigation	Good safety profile Broad immunomodulatory effects Paracrine neuroprotection Multiple delivery routes	Poor CNS homing Donor variability Modest efficacy Potential tumorigenicity
Neural Stem Cells (NSCs)	Fetal tissue, Fetal or adult CNS tissue, pluripotent stem cells (iPSCs), Human embryonic stem cells (hESCs)	Differentiation into neurons/oligodendrocytes, neuroprotection, remyelination, neurotrophic support	Intrathecal, intracerebral	Preclinical and early-phase clinical studies	Direct cell replacement Strong remyelination potential Relevant for progressive MS	Invasive delivery Low graft survival Immunogenicity/tumorigenicity risks Limited migration
Regulatory T Cells (Tregs)	Autologous peripheral blood expanded ex vivo	Restoration of immune tolerance; suppression of autoreactive T cells	Intravenous	Phase I–II trials	Highly specific immunoregulation Autologous and safe Avoids immunoablation	Short persistence Complex manufacturing Small early trials
Oligodendrocyte Progenitor Cells (OPCs)	Fetal/adult CNS, iPSCs, hESCs	Remyelination via differentiation into oligodendrocytes	Intranasal, intracerebral	Preclinical	Direct remyelination strategy Strong myelin-forming capacity	Poor engraftment Sensitive to inflammation/steroids Invasive procedures Early-stage evidence only
Induced Pluripotent Stem Cells (iPSCs)	Reprogrammed adult somatic cells	Differentiation into NSCs/OPCs; neuroregeneration and immunomodulation	Intravenous or intrathecal	Preclinical	Unlimited autologous source Ethical advantages over hESCs Can generate diverse therapeutic lineages	Genetic instability Tumorigenicity risk Complex manufacturing Regulatory challenges
Extracellular Vesicles (EVs)	Secretome of MSCs or other stem cells	Paracrine signaling; immunomodulation; neuroprotection; delivery of bioactive molecules	Intravenous, intranasal, intrathecal	Preclinical	Cell-free → safer profile Can cross BBB Scalable and stable	Early development stage Unclear biodistribution No proven clinical efficacy

## Data Availability

Not Applicable.

## Supplementary Materials

The following supporting information can be downloaded at: https://www.mdpi.com/article/10.3390/pharmaceutics18010030/s1, PRISMA-ScR 2018 Checklist. Ref. [93] cited in Supplementary Materials.
